# Effective attention-based network for syndrome differentiation of AIDS

**DOI:** 10.1186/s12911-020-01249-0

**Published:** 2020-10-15

**Authors:** Huaxin Pang, Shikui Wei, Yufeng Zhao, Liyun He, Jian Wang, Baoyan Liu, Yao Zhao

**Affiliations:** 1 Beijing Jiaotong University,China, No.3 Shangyuancun, Haidian District, Beijing, 100044 China; 2 Institute of Basic Research in Clinical Medicine/National Data Center of Traditional Chinese Medicine, China Academy of Chinese Medical Sciences, No.16 South Street,Dongzhimen,Dongcheng District, Beijing, 100700 China; 3 China Academy of Chinese Medical Sciences, No.16 South Street,Dongzhimen,Dongcheng District, Beijing, 100700 China

**Keywords:** Traditional chinese medicine, Machine learning, Syndrome differentiation, AIDS

## Abstract

**Background:**

Syndrome differentiation aims at dividing patients into several types according to their clinical symptoms and signs, which is essential for traditional Chinese medicine (TCM). Several previous works were devoted to employing the classical algorithms to classify the syndrome and achieved delightful results. However, the presence of ambiguous symptoms substantially disturbed the performance of syndrome differentiation, This disturbance is always due to the diversity and complexity of the patients’ symptoms.

**Methods:**

To alleviate this issue, we proposed an algorithm based on the multilayer perceptron model with an attention mechanism (ATT-MLP). In particular, we first introduced an attention mechanism to assign different weights for different symptoms among the symptomatic features. In this manner, the symptoms of major significance were highlighted and ambiguous symptoms were restrained. Subsequently, those weighted features were further fed into an MLP to predict the syndrome type of AIDS.

**Results:**

Experimental results for a real-world AIDS dataset show that our framework achieves significant and consistent improvements compared to other methods. Besides, our model can also capture the key symptoms corresponding to each type of syndrome.

**Conclusion:**

In conclusion, our proposed method can learn these intrinsic correlations between symptoms and types of syndromes. Our model is able to learn the core cluster of symptoms for each type of syndrome from limited data, while assisting medical doctors to diagnose patients efficiently.

## Background

Syndrome diffetentiation in Traditional Chinese Medicine (TCM) syndromes is a method of classifying the whole functional status summarized by clinical symptoms of different individuals during a period of illness. In TCM, this is one of the crucial aspects to study syndromes and plays a guiding role in clinically individualized diagnosis and dialectical treatment of TCM. In recent years, researchers have intensively studied the efficacy of TCM in the treatment of AIDS [1–3]. Many clinical practices and data proved that TCM had made surprising progress in reducing the HIV viral load in the blood and relieving patients’ clinical symptoms and improving quality of life [4–11]. These advances are mainly attributed to the fact that TCM practitioners classified AIDS patients with syndromes and cured them with Chinese medicine treatments. It can be seen that syndrome classification is of great significance in the field of TCM.

Differentiation is at the core of TCM and sets the precondition that ensures efficacy. Usually, the approaches for classifying the syndromes in TCM, which include multivariate statistical methods, machine learning, neural networks, and other methods introduced into the study, have resulted in an extensive set of scenarios. Among the group of multivariate statistical methods, cluster analysis is one of the fundamental statistical methods. It is widely used in syndrome differentiation due to the trait that it avoids the negative impacts of individual subjectivity. Researchers such as Martis and Chakraborty tried to classify and explore the principles for the cause of arrhythmia disease [12]. As the representative of machine learning algorithms, support vector machine (SVM) is one of the most commonly used diagnostic classification models, and has been used by researchers such as Ekiz et al. to diagnose heart disease through SVM [13]; by Chen et al. to diagnose hepatitis disease [14] and by Zeng et al. to diagnose Alzheimer’s disease [15]. Pang and Zhang tried to use a naive Bayesian network to reveal the connection between abnormal tongue appearances and diseases in a particular population [16]. In recent researches, deep learning models have been widely used to diagnose diseases. Models such as noisy deep dictionary learning [17], deep belief networks (DBN) [18], and long-short term memory network (LSTM) [19] have achieved better results.

Although these methods have achieved significant improvement in syndrome classification, they remain far from being satisfactory. First, when all symptoms are used equally for classification, uncorrelated symptoms can have excessive disturbing influences. In such cases, most of these algorithms cannot quite figure out what are the representative symptoms in one type of syndromes for diverse diseases. Moreover, because of the obvious differences between the diseases, there is no unified classification model for all illnesses. Due to the particularities of AIDS, most patients suffer from multiple diseases at the same time, and the clinical symptoms are various and complex [7, 9]. These causes make it relatively difficult to judge patient’s syndrome type and to define suitable treatment protocols.

To tackle the above-mentioned limitations, here we propose an attention-based MLP framewor (ATT-MLP). Our model relies on an attention mechanism that directly draws the global dependencies of the inputs [20]. We build feature-level attention with a hidden layer over multiple symptoms, which is expected to dynamically reduce the weights of noisy symptoms. Finally, the sequence where all symptom features are weighted by feature-level attention is fed into the MLP to discriminate types of syndrome. In contrast to other algorithms, the attention mechanism is trained to capture the dependencies that make significant contributions to the work, regardless of the distance between the elements in the sequence. Another major advantage of attention is that computing the attention only requires matrix multiplication, which is highly parallelizable and easily realized [21]. With the proposed simple yet effective ATT-MLP, we evaluated our model on a real-world AIDS dataset that integrates data from multiple clinical units to provide a comprehensive view of syndrome differentiation and medication patterns of AIDS [22]. In term of accuracy-score, our experimental results outperformed traditional algorithms by 13.4%. At the same time, our model also selected symptoms associated with the type of syndrome that is in line with the actual clinical situation.

## Related work

The rapid adoption and availability of disease treatment records using TCM have enabled new investigations into data-driven clinical support. The broad goal of these studies is to learn from the datasets of patients’ records and provide personalized treatment to them. Here, we provide a brief overview of work specifically in diagnosis and treatment of diseases as well as applications of attention-base models in different fields.

### Diagnosis and treatment of diseases

Currently, some innovative classification techniques apply to quantitative syndrome analysis. Li and Yan et al. used the k-Nearest Neighbor (k-NN) model for classification of hypertension [23]. Bayesian networks have been used to make quantitative TCM diagnosis of diseases [24]. Some studies [25–27] have also used SVM to classify disease data with certain low feature dimensions. These algorithms have achieved good results in both disease diagnosis and re-classification tasks. However, the degree of syndrome correlation with symptoms in the data set of AIDS is different. The traditional k-NN algorithm [22] that assigns the same weight to each dimension or symptom does not fit the task well. The dataset has a high data dimension and the symptoms of patients are sparse. An SVM based on distance metric classification also fails to achieve ideal results. The Bayes classification algorithm based on prior probability can reveal its inadequacies when handling high-dimensional features. Artificial neural networks (ANN) boasts large-scale distributed parallel processing, nonlinear, self-organizing, self-learning, associative memory, and other excellent features. ANN has been used to achieve many gratifying results, and some researchers [28, 29] have begun to use neural networks to conduct an exploratory study on the classification of diseases and syndromes according to TCM. Thanks to these efforts, the effectiveness of the diagnosis and classification of diseases has been significantly improved.

### Attention applications

Attention-based models have attracted much interest from researchers recently. The selectivity of attention-based models allows them to learn alignments between different modalities. Such models have been used successfully in several tasks. Minh et al. [30] used recurrent models and attention to facilitate the task of image classification. Ling et al. [31] proposed an attention-based model for word embedding, which calculates an attention weight for each word at each possible position in the context window. Parikh et al. [32] utilized attention for the task of natural language inference. Lin et al. [33] proposed sentence-level attention for sentence embedding and applied them to author profiling, sentiment analysis, and textual entailment. Paulus, Xiong, and Socher [34] combined reinforcement learning and self-attention to capture the long-distance-dependencies nature of abstractive summarization. Vaswani et al. [35] carried out the first study to construct an end-to-end model with attention alone and reported state-of-the-art performance in machine translation tasks. Our work follows this line and applies attention to learning long-distance dependencies. To the best of our knowledge, this is the first effort to adopt an attention-based model for TCM syndrome differentiation.

## Methods

### Frame structure

**Fig. 1:**
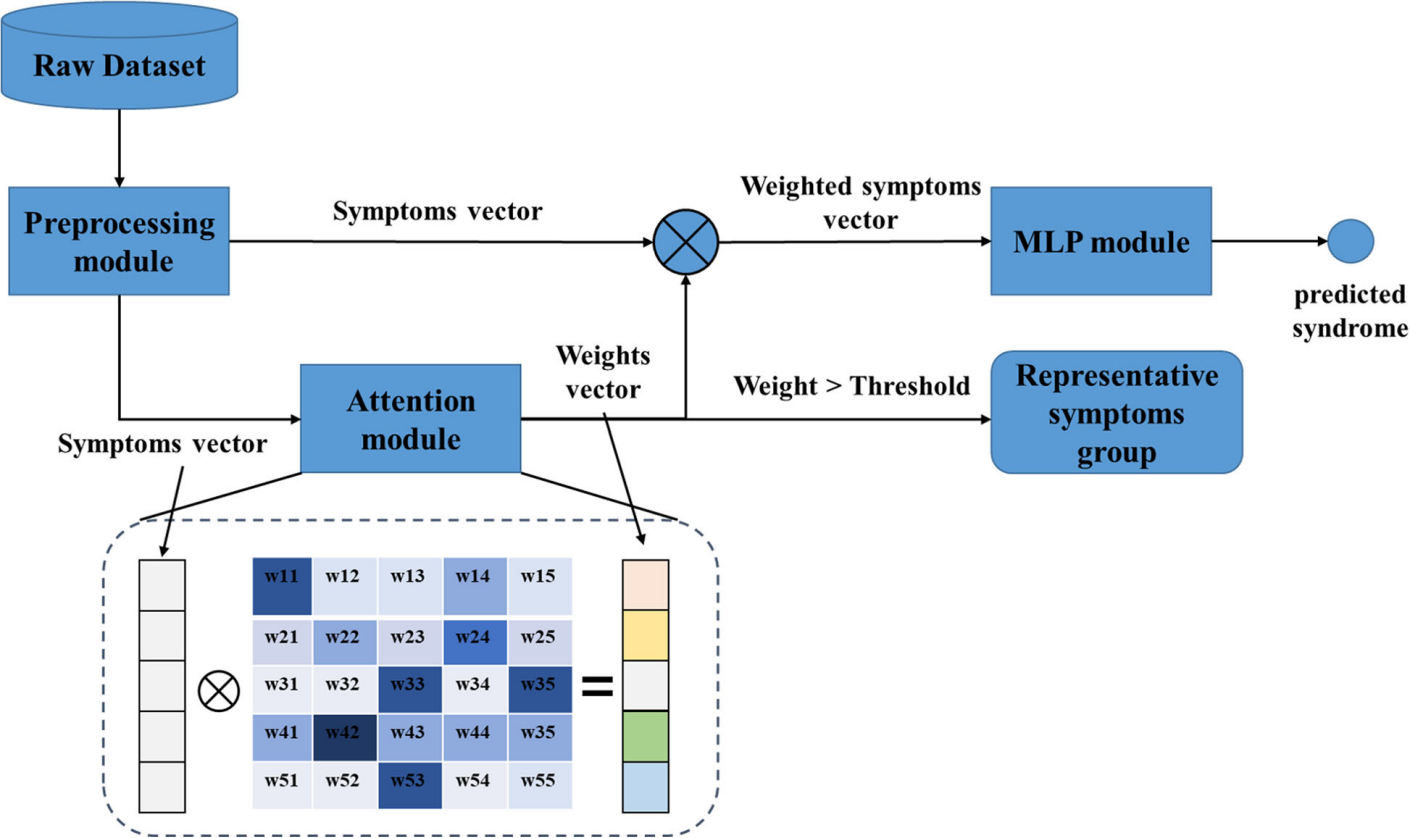
Architecture of the ATT-MLP model used for syndrome classification. The original symptoms sequence is taken as the only input for ATT-MLP

Three main functional modules are included in the proposed method. They are setting the initial symptom feature vectors and syndrome label vectors, calculating attention weights for different symptom characteristics under different labels, and iterative training of the classification model. The framework of the proposed method is illustrated in Fig. 1.

### Attention-based framework

The definition of the symbols is given here to explain the attention-based model. The patient set is *P*={*p*_*nm*_,*n*=1,...,*N*,*m*=1,...,*M*},where N is the total number of patients and M is the total number of the patients’ symptoms. The syndrome type of AIDS is *S*={*c*_*i*_,*i*=1,...,*K*},where K is the total number of syndrome types. The purpose of the attention-based model is to obtain different symptom weights based on the patient’s own symptoms and syndrome characteristics. Then, the attention weight is realized by optimizing the function, which is shown in Eq. (1).

1 $$  \mathbf{E_{ns}}=\tanh\left(A \cdot \boldsymbol{p}_{\boldsymbol{n}}\right)  $$

Where *p*_*n*_ is the query vector of a patient’s symptoms, A is a weighted matrix. *E*_*ns*_is the attentional vector that scores how well all symptoms and the syndrome type match. We selected the non-linear form *tanh*, which achieves the best performance when considering different alternatives.

Then, we chose the *softmax* function to normalize the attentional vector, which is shown in Eqs. (2)and (3).

2 $$\begin{array}{*{20}l} \mathbf{W_{ns}}=\mit{softmax}\left(\mathbf{E_{ns}}\right)  \end{array} $$

3 $$\begin{array}{*{20}l} w_{i} = \frac{\exp (e_{i})}{{\sum\nolimits}_{i=1}^{M} \exp(e_{i})}  \end{array} $$

Where *W*_*ns*_ is the normalized weight vector, *e*_*i*_ is the i-*th* weight probability corresponding to the i-*th* symptom, and *w*_*i*_ is the normalized weight value.

The final output is the patient’s feature vector based on attention weight as in Eq. (4):

4 $$  \mathbf{P^{*}_{n}}=\mathbf{P_{n}} \cdot \mathbf{W_{ns}}  $$

### Multilayer perceptron

We used a multilayer perceptron(MLP) as the last syndrome classifier. The MLP is a type of ANN composed of multiple hidden layers, where every neuron in layer *i* is fully connected to every other neuron in layer *i*+1. Typically, these networks are limited to a few hidden layers, and the data flows only in one direction, unlike recurrent or undirected models. The input is a one-dimensional vector that is consistent with the format of our data set. In our proposed method, the structure of MLP consists of three layers, which are input, hidden and output. Each hidden unit computes a weighted sum of the outputs from the input layer, followed by a nonlinear activation *σ* of the calculated sum as in Eq. (5).

5 $$  h_{i} = \sigma\left(\sum\limits_{j=1}^{d} p_{rj}w_{ij}+b_{ij}\right)  $$

Here, *d* is the number of units in the input layer, which is also the number of symptom features. *p*_*rj*_ is the weighted value of the j-*th* symptom of the n-*th* patient. And *w*_*ij*_ and *b*_*ij*_ are the weight and bias terms associated with each *p*_*rj*_. Traditionally, *sigmoid* or *tanh* are chosen as the nonlinear activation functions, but we utilized rectified linear units (ReLU) to get good results in our model.

It is worth noting that the attention-based model and MLP were trained at the same time. We used the backpropagation algorithm to update the parameters in these models. To verify the performance of the model, the desired significance level is set to 0.05.

## Results

### Dataset of AIDS in TCM

In this study, we used data from the TCM pilot project for treating AIDS, which contains over 12,000 patients’ records from over 17 provinces covering the years from 2004 to 2013. The ethics committees of the Institute of Basic Research in Clinical Medicine, China Academy of Chinese Medical Sciences, granted exempt status for this study and also waived the need for informed consent. The dataset recorded the personal and disease history, symptoms, syndromes, and treatment strategies provided by certified senior medical doctors. The symptom group contains a total of 93 symptoms. There are seven types of syndrome for these patients in the dataset according to the AIDS syndrome diagnostic criteria, which includes (S1) phlegm-heat obstructing the lung and accumulation of heat toxin; (S2) deficiency of both qi and yin in the lung and kidney; (S3) stasis of blood and toxin due to qi deficiency; (S4) hot in the liver with accumulation of damp toxin; (S5) stagnation of qi, phlegm and blood; (S6) deficiency of spleen and stomach with retention of damp; and (S7) qi deficiency with kidney yin deficiency. From the entire AIDS dataset, we selected 10,910 cases based on the inclusion criteria. The criteria for inclusion of patients in this study were(1) age over 18; (2) complete TCM syndrome diagnosis record completed; (3) explicit symptoms; (4) patients presenting with at least two symptoms; and (5) patients providing informed consent. Of the 10,910 patients, 6,248 patients were male(˙57.27% with a mean age of 41.53 ±9.47), and 4,662 patients were female(˙42.73% with 36.39 ±8.54).

### Experimental and evaluation metric

In practice, we find that certain symptoms are different in their relevance and importance for these syndromes. Hence, we build a model for each type of syndrome. In the model, we used all positive samples and some randomly selected samples with other syndromes as the negative samples in the whole dataset to ensure that the ratio of positive to negative was about 1:2. All models were trained and tested through a 10-fold cross-validation method. Meanwhile, and the average of multiple test scores was recorded in these tables. To check the robustness of our model, we provided the prediction results with the five indicators: Accuracy, Sensitivity(Recall), Specificity, Matthews correlation coefficient(MCC), and Area Under the ROC curve(AUC).

### Selected symptoms based on attention

We used the proposed model to select representative symptoms to characterize the features of each AIDS syndrome. The selected representative symptoms for seven AIDS syndromes are shown in Table 1.

**Table 1:** Selected representative symptoms for seven AIDS syndrome types, Here, we have selected symptoms whose attention weight is greater than 0.8 to characterize the features of each AIDS syndrome

**Syndrome**	**Selected representative symptoms**	**Accuracy(%)**
S1	white tongue coating; red tongue; string-taut pulse; cough; fever;	85.2
	headache; chest pain; rash or herpes; self-sweating; itchy skin; dizziness	
S2	scanty coating; fine vein; rapid pulse; dry coating; low fever;	86.4
	night sweating; fatigue; pale complexion; itching in the skin; cough	
S3	dark purple tongue; white coating; unsmooth pulse; fatigue; fever;	83.5
	dyspnea on exertion; alopecia; muscle ache; joint pain; dark and gloomy complexion	
S4	red tongue; greasy coating; slippery pulse; herpes; skin ulcer;	87.2
	itching in the skin; ulcer in the tongue; asthma	
S5	pink tongue; thin coating; string-taut pulse; lack of appetite;	85.9
	weight loss; cold sweat; anorexia; scrofula bump	
S6	pale tongue; yellow and white tongue coating; thick coating; greasy coating; deep pulse;	84.2
	slippery pulse; diarrhea; abdominal pain; nausea; tired soreness; fever; prolapse	
S7	thin tongue; grey and black coating; thin coating; weak pulse;	**87.6**
	fatigue; anorexia; dyspnea on exertion; skin ulcer	

According to Table 1, we could find that ATT-MLP performs well in the task of selecting representative symptoms to characterize each syndrome and all accuracy of syndromes are more than 80%. On closer inspection, the accuracy of S4 and S7 syndromes exceed 87.0%. And the S7 syndrome got the highest accuracy, reaching 87.6%. In addition, this worst performance (S3) is still 83.5%. Overall, our model automatically diagnosed the syndrome type of AIDS patients by selecting symptoms, and its performance was close to the level of most clinicians. Secondly, through observing Table 1, we could also find out that some symptoms appeared in the symptom group of multiple syndromes at the same time. For example, fever occurring in S1, S3, and S6; cough appearing in both S1 and S2, and slippery pulse appearing in both S4 and S6. White tongue coating was included in S3 and S6. More detailed reasons are given for above-mentioned conditions. On one hand, the problems in different parts of the body may show the same symptoms. On the other hand, a same symptom plays different roles in different syndrome types. Combined with Table 1 and compared with S3, symptom fever plays a more primary role in syndrome S1. Diagnosing syndrome accurately needs to integrate several symptoms’ information. In order to explore the contribution of each symptom to syndrome type and disease diagnosis, more details and further discussions about symptoms’ importance are discussed in the next section-Symptom Weights for Different Syndrome types.

### Symptom weights for different syndromes

**Fig. 2:**
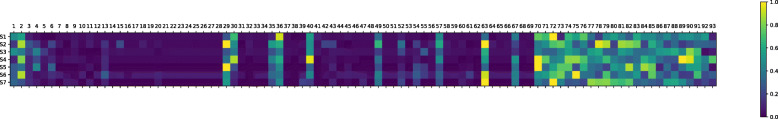
Heat maps of symptoms with attention weights for seven syndromes plotted by symptoms on the horizontal axis and syndromes on the vertical. Each cell shows the relevance percentage of symptoms for each syndrome

For each syndrome type, we collected the attention weight vectors for all patients within them and subjected them to normalization to obtain Fig. 2. The rows of the matrix in the figure represent seven syndrome types of the AIDS dataset and each column represents the attention weight values for one symptom.

We can see that for different syndromes, the type and number of symptoms on which the model focused were significantly different from Fig. 2. For instance, the number of symptoms of concern for S3 and S5 is more than for S7. Focusing on the local details of the matrix, some symptoms occupy a higher weight in certain syndrome types, We can find that the importance of red tongue(2) is different for seven syndrome type: the higher importance for S2 and S6, compared with S1, S4, and S5. The representative symptom white tongue coating(29) also is closely related to S2 and S5, and selected as their key symptom in Table 1. Nevertheless, only the syndrome S1 has a greater dependence on the symptom-scanty coating(36), and the weight between S1 and scanty coating is over 0.9. there another noteworthy phenomenon is that though the weights of two symptoms-cough(72) and fatigue(73) are high in all of the syndromes, they just are regarded as generalized features, not typical symptoms. These cases are in line with the actual situation.

On other hand, the current model can focus on different dimensional symptoms, and the distances of these symptoms are relatively distant from the global perspective of the map. This implies that our model has prominent advantages in dealing with high-dimensional information in medical diagnosis. The heat map in Fig. 2 also shows that our model not only makes use of local symptom information for diagnosis and prediction but also combines global information to diagnose the patient’s syndrome category. It is similar to the disease diagnosis method used by clinical experts, indicating that the internal mechanism of ATT-MLP is to realize the goal of predicting syndrome by studying and applying some real human experience.

### Comparison

Tables 2 and 3 recorded the performance scores of 5 algorithms, measured with indicators including: Accuracy, Sensitivity(Recall), Specificity, MCC and AUC. The best result for each column was highlighted. Overall, our framework based on the attention mechanism significantly improved performance over the other four models in most indicators on each of the AIDS syndromes. The average of the Accuracy of syndrome differentiation based on ATT-MLP was 85.7% and the average of sensitivity and specificity reaches 74.6% and 91.3%, which were more than 10% higher than other models. However, the SVM had worse performance in the predicted task, the main reason was that the SVM classifier used all the symptoms of patients, but without considering and selecting some key symptoms and without using a fusion of different symptom information to classify syndrome type. Through analyzing all the experimental results of syndrome type, we could find that the classification effect of the five models for S4 and S7 were significantly better than for other syndromes, which illustrates that the symptomatic composition of both types of syndromes was relatively fixed and less affected by other symptoms. Obviously, the proposed model also achieved the best performance, the accuracy and sensitivity scoring 87.6%, and 83.7% in S7. These results further indicated that the key symptoms of S7 were obvious and easy to find and capture.

**Table 2:** Performance comparison of our proposed model and traditional methods on dataset. The A, Se, and Sp mean Accuracy, Sensitivity, and Specificity in the table

**Methods-(%)**															
**Syndromes**	**Naive Bayes**			**SVM**			**RF**			**MLP**			**ATT-MLP**		
	**A**	**Se**	**Sp**	**A**	**Se**	**Sp**	**A**	**Se**	**Sp**	**A**	**Se**	**Sp**	**A**	**Se**	**Sp**
S1	73.8	60.2	80.6	70.4	58.7	76.3	81.1	72.7	85.2	77.9	65.3	84.2	85.2	77.1	89.2
S2	78.9	68.3	84.3	71.7	62.3	76.4	84.5	76.5	88.5	77.6	67.5	82.7	86.4	72.2	93.4
S3	69.9	61.1	74.3	69.0	59.4	73.9	83.6	73.9	88.5	76.2	65.9	81.3	83.5	66.5	92.0
S4	79.8	72.9	83.2	72.9	63.7	77.5	85.3	81.8	87.1	82.6	74.3	86.7	87.2	79.6	91.0
S5	66.7	57.1	71.4	71.5	61.7	76.4	81.4	80.5	81.8	66.7	55.2	72.4	85.9	74.8	91.4
S6	77.2	68.4	81.6	74.5	65.6	79.0	85.2	76.7	89.5	72.8	62.6	77.9	84.2	68.0	92.2
S7	77.7	70.1	81.4	76.0	66.2	80.9	80.2	71.0	84.8	78.1	69.2	82.5	87.6	**83.7**	89.6
**Avg.**	74.8	65.4	79.5	72.3	62.5	77.2	83.0	76.1	86.5	76.0	65.7	81.1	85.7	74.6	91.3

**Table 3:** The performance comparison of robustness and generalization of multiple models and the independent test results of ATT-MLP are presented. The MCC and AUC are for Matthews correlation coefficient and Area Under the ROC curve in the table

**Methods**											
**Syndromes**	**Naive Bayes**		**SVM**		**RF**		**MLP**		**ATT-MLP**		
	**MCC**	**AUC**	**MCC**	**AUC**	**MCC**	**AUC**	**MCC**	**AUC**	**MCC**	**AUC**	***P***
S1	0.41	0.63	0.34	0.69	0.58	0.74	0.50	0.71	0.67	0.79	<.001
S2	0.53	0.70	0.38	0.72	0.65	0.74	0.50	0.72	0.69	0.79	<.001
S3	0.34	0.64	0.32	0.68	0.63	0.75	0.47	0.73	0.62	0.74	<.001
S4	0.55	0.76	0.40	0.73	0.68	0.78	0.61	0.74	0.71	0.82	<.001
S5	0.28	0.60	0.37	0.72	0.60	0.73	0.27	0.70	0.68	0.78	<.001
S6	0.49	0.68	0.44	0.75	0.67	0.80	0.40	0.74	0.63	0.76	<.001
S7	0.51	0.71	0.47	0.74	0.56	0.83	0.51	0.76	0.73	**0.84**	<.001
**Avg.**	0.44	0.68	0.39	0.72	0.62	0.77	0.47	0.73	0.67	0.79	

Because the number of positive and negative samples are unbalanced, we employed MCC and AUC indicators to measure the robustness of our model. Meanwhile, and we performed ATT-MLP over the dependent test to measure the reliability of cross-validation results. Judging from Table 3, Random Forest(RF) and ATT-MLP model have compared results. The MCC’s score was above 0.5 and the average of AUC’s result reached 0.77 and 0.79 for RF and ATT-MLP. This showed that facing the problem of unbalanced samples, our model could deal with it by learning the internal mapping between symptoms and syndrome. The P-values of the independence test of our model were all less than 0.001, which verified the truthfulness of the experimental results of our model and indicated that the model could extract effectively the representative symptom group of syndromes.

It is noteworthy to point out that the MLP model is our baseline model. Viewing Table 3, we see that the MLP model based on the attention mechanism shows a significant improvement over the performance of the original model. There is enough evidence to show that the attention mechanism framework plays an important role in the task of capturing key symptoms. Whihout changing the structure of the data itself, the attention mechanism can help MLP to optimize and classify in the right direction by scoring the symptoms.

All models had poor classification performance for S3 and S5, especially in the indicator-Sensitivity score, compared to the other five syndromes. It means that by only relying on the dataset, these models can’t find and learn the key symptom groups of S3 and S5. For our model(ATT-MLP), the difficulties for learning and mining the pivotal symptom groups of S3 and S5 syndromes are objective subsistent. The initial conjecture about the problem is that the symptoms of these patients labelled as the two syndromes (S3 and S5) are complex and diverse and that the interaction between symptoms is complicated. In order to test this hypothesis, we conducted the following experiment: the evaluation and measurement of the number of symptoms related to the syndrome.

### Number of syndromes & metric

**Fig. 3:**
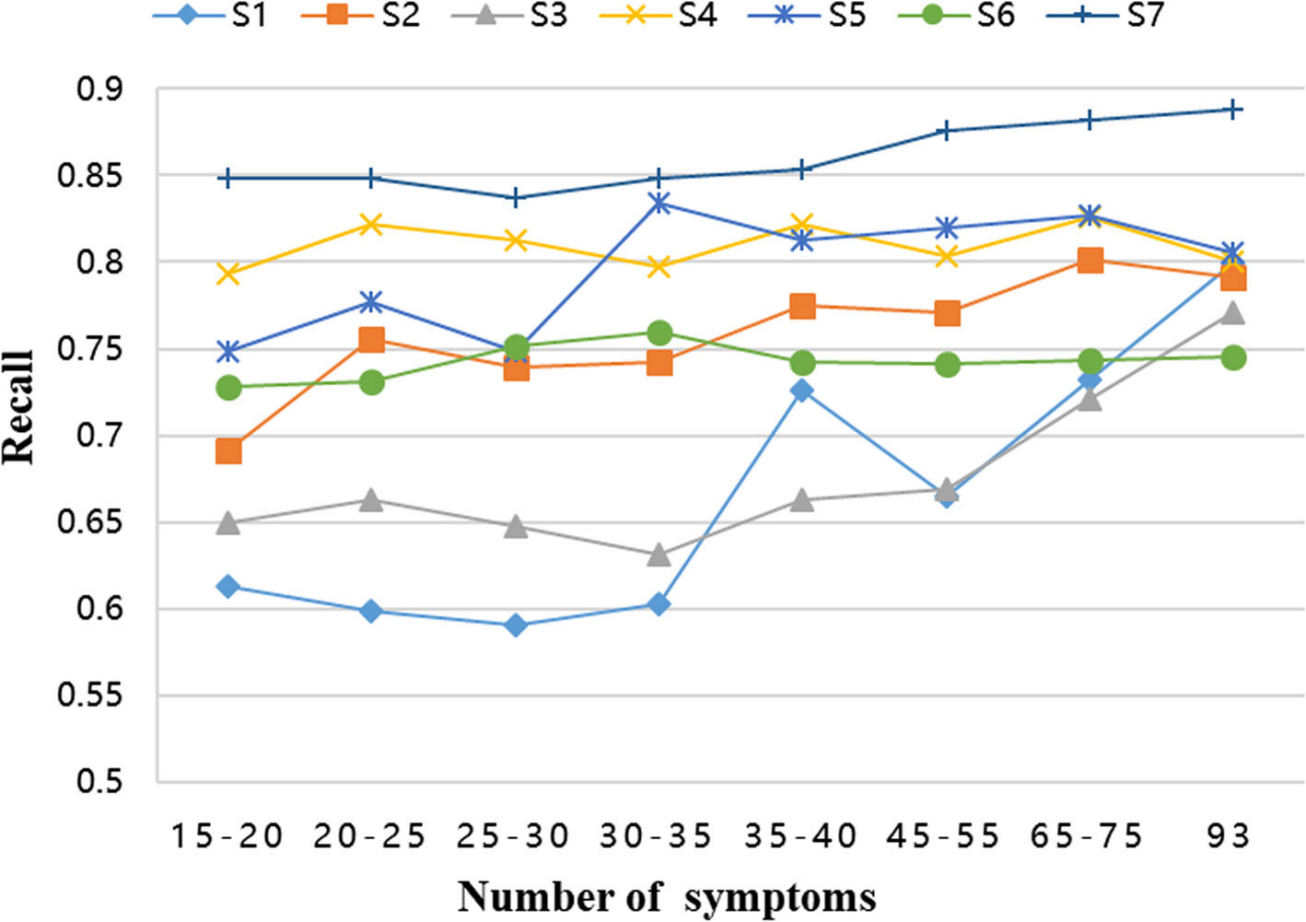
Performance comparison in the case of different numbers of representative symptoms for seven syndromes

In order to verify whether the symptoms selected by the proposed model based on the attention mechanism are the key symptoms of the certain syndrome, we verified the results. Firstly, we used the attention framework to score all symptoms and to remove some symptoms according to different score thresholds. Then the remaining symptoms were re-scored to classify syndromes. Recording model classification performance indicators with different thresholds. The changes in these indicators are shown in Fig. 3.

**Fig. 4:**
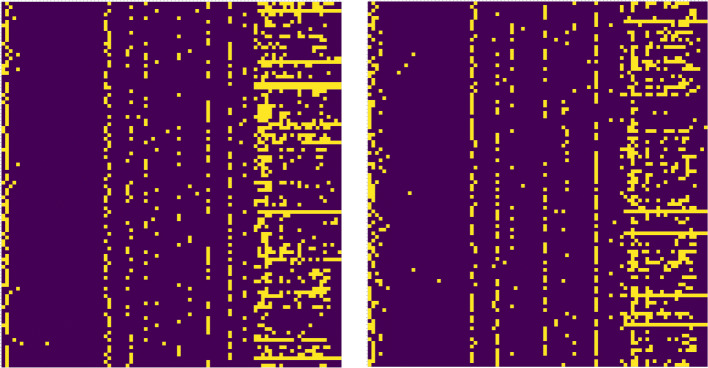
The sample cases hot map of syndromes S1-(a) and S3-(b). the symptoms and sample index severally are shown on the horizontal and the vertical axis

By observing these figures, we can clearly see that as the number of selected symptoms decreases, there is no significant decline in the classification performance of our model. As to syndromes-S4 and S7, with the number of symptoms decreasing, our model keeps high-level score in the accuracy rate of diagnosis, which suggests that their main symptom groups remain stable and our model can efficiently extract their core symptoms from some limited samples. However, through observation of Fig. 3, the classification effects of S1 and S3 syndromes were greatly affected by the change in the number of symptoms, and the change of the index was more than 15%. One convictive explanation for these phenomena is that the combinations of primary symptom for different syndromes are diverse and the association between the symptoms is complex. We further randomly selected 100 samples labelled S1 and S3 separately, shown in Fig. 4. For syndrome-S1, main symptoms such as the red tongue(2), yellow coating(30), string-taut pulse(70), and fever(71) are relatively obvious to be seen, but other symptoms are difficult to summarize. As for syndrome-S3, white coating(29) and thready pulse(63) is easily detected. In this case, a single framework does not fit well into these clinical diagnostic methods. We need to combine other methods to do more comprehensive researches.

Secondly, we can also find another fact from these figures. As the selected symptoms decrease, all the indicator values decrease slightly. This illustrates that while our model eliminates the effects of irrelevant symptoms, it is also possible to delete certain key symptoms unintentionally or to sever the relationships between certain features. Since these results to have a small decrease in model performance, we will conduct further exploration and research of these matters in follow-up work.

## Discussion

In this paper, we proposed an ATT-MLP-based syndrome classification model. Our model can diagnose the syndrome of AIDS patients effectively, and select representative symptoms of each syndrome. This provides new ideas and methods for the establishment of a TCM syndrome differentiation model for patients with AIDS. Our proposed model shows good performance in the classification of the seven syndromes in the dataset and gives an average accuracy of 85.7%, an average sensitivity (recall) rate of 74.6%, and an average specificity of 91.3%. The results of these classification experiments are superior to what has been obtained using more conventional classifiers in previous studies.

There are two advantages to the proposed model. First, compared with other syndrome differentiation models, our model can accurately select representative features to characterize explicitly the characteristics of the AIDS syndrome. This provides an objective basis for TCM syndrome differentiation, which often relied on empirical medicine. Second, our model can also assign reasonable weights to the selected symptoms. For the same symptom, the weight is different for different syndromes, which means that only the more heavily weighted symptoms play a key role in the diagnosis of a given syndrome. This can help doctors to develop treatments that are appropriate for their patients.

Due to the complexity of AIDS, some patients may have several AIDS syndromes. However, current attention-based models are not good at handling the multi-label task classification. In the future, we will consider abandoning this model, which scores the symptoms individually. Instead, we will explore the effect of the degree of association between the symptoms on the diagnosis of the syndrome. Then we could use the efficient deep learning framework to build a more complete syndrome differentiation model.

## Conclusions

In conclusion, our proposed method can learn these intrinsic correlations between symptoms and syndromes. As a matter of fact, the relevant information was summarized by experts with rich clinical experience. These experiments demonstrate that our model can use the attention mechanism to select representative symptoms for each syndrome and improve the diagnosis accuracy of patients’ syndromes.

## Data Availability

This study was based in part on data from the TCM pilot project for treating AIDS, provided by the Institute of Basic Research in Clinical Medicine, and was managed by China Academy of Chinese Medical Sciences. The datasets are available from the corresponding author on reasonable request.

## Abbreviations

TCM: Tradition chinese medicine
ATT-MLP: Multilayer perceptron model with the attention mechanism
AIDS: Acquired immune deficiency syndrome
HIV: Human immunodeficiency virus
SVM: Support vector machine
DBN: Deep belief networks
LSTM: Long-short term memory networks
ANN: Artificial neural network
